# Time Sequential Single-Cell Patterning with High Efficiency and High Density

**DOI:** 10.3390/s18113672

**Published:** 2018-10-29

**Authors:** Yang Liu, Dahai Ren, Xixin Ling, Weibin Liang, Jing Li, Zheng You, Yaxiaer Yalikun, Yo Tanaka

**Affiliations:** 1 State Key Laboratory of Precision Measurement Technology and Instruments, Tsinghua University, Beijing 100084, China; ly-15@tsinghua.org.cn (Y.L.); xixin.ling@ptpcapital.com (X.L.); liang-wb12@tsinghua.org.cn (W.L.); lijing18@mails.tsinghua.edu.cn (J.L.); yz-dpi@tsinghua.edu.cn (Z.Y.); 2 Center for Biosystems Dynamics Research (BDR), RIKEN, 1-3 Yamadaoka, Suita, Osaka 565-0871, Japan; yaxiaer.yalikun@riken.jp (Y.Y.); yo.tanaka@riken.jp (Y.T.); 3 Division of Materials Science, Nara Institute of Science and Technology, 8916-5 Takayama-cho, Ikoma, Nara 630-0192, Japan

**Keywords:** cell patterning, cell trapping, single-cell analysis, microfluidics, lab-on-a-chip

## Abstract

Single-cell capture plays an important role in single-cell manipulation and analysis. This paper presents a microfluidic device for deterministic single-cell trapping based on the hydrodynamic trapping mechanism. The device is composed of an S-shaped loop channel and thousands of aligned trap units. This arrayed structure enables each row of the device to be treated equally and independently, as it has row periodicity. A theoretical model was established and a simulation was conducted to optimize the key geometric parameters, and the performance was evaluated by conducting experiments on MCF-7 and Jurkat cells. The results showed improvements in single-cell trapping ability, including loading efficiency, capture speed, and the density of the patterned cells. The optimized device can achieve a capture efficiency of up to 100% and single-cell capture efficiency of up to 95%. This device offers 200 trap units in an area of 1 mm^2^, which enables 100 single cells to be observed simultaneously using a microscope with a 20× objective lens. One thousand cells can be trapped sequentially within 2 min; this is faster than the values obtained with previously reported devices. Furthermore, the cells can also be recovered by reversely infusing solutions. The structure can be easily extended to a large scale, and a patterned array with 32,000 trap sites was accomplished on a single chip. This device can be a powerful tool for high-throughput single-cell analysis, cell heterogeneity investigation, and drug screening.

## 1. Introduction

Average-response-based cell analysis techniques have been widely adopted in both biological research and clinical diagnosis. However, several malign mutations that cause maladies such as leukemia [1], liver cancer [2], and Alzheimer’s disease [3] usually start from a rare subpopulation of cells. The average-response-based analysis often fails to reveal some abnormal lesions because of the intrinsic drawbacks of this method, such as omission of “error” or “noise” signals [4,5]. The single-cell analysis focuses on the unique response of the subpopulation cells exposed to different stimuli [6]. It is also suitable for the collection and analysis of such cells, which enables early diagnoses of many diseases, selection of patient-defined therapies, and further study of these abnormal cells [7,8].

Single-cell capture and separation with high efficiency are the fundamental and imperative requirements for single-cell analysis. Conventional methods such as dielectrophoretic [9,10], magnetic [11,12], optical [13], acoustic [14], flow cytometry [15,16] and immune [17] methods have been used to capture cells. However, there are still some obstacles in using these methods to simultaneously achieve high efficiency, high accuracy, and large-scale patterning, together with little mechanical damage, low cell loss, and low cost. For example, the dielectrophoresis method causes damage to cell viability because of dielectric forces and heat; the flow cytometry method has a high throughput, but causes a potential mechanical damage to the cells because of high shear force.

Hydrodynamic cell patterning methods are usually favorable because of their advantages of low cost, relatively high throughput, and resistance to unwanted influence of various factors [18,19]. Most methods that use passive trapping mechanisms have a limit on capture efficiency and manipulation of the trapped cells after their capture [20,21,22] with severe cell loss. Microwell array methods are typically used for single-cell patterning, and most of them take advantage of gravity to trap the cells. Many studies have been conducted in which a high single-cell capture efficiency has been achieved with the microwells densely patterned on a large scale; the optimized design enables a capture efficiency of up to 70% [23,24]. The capture efficiency is limited by the possibility of the cells being randomly distributed in the channels before flowing into the microwells. Moreover, it is difficult to recover the cells from the microwells for further manipulation.

Another typical method is using fluid flow to direct the cells into the trap sites [25,26]. For example, Zhang [21] proposed a microfluidic device with hook arrays along the sidewalls of the channels to capture the cells with a single-cell capture efficiency of more than 95%. However, it is necessary to have more cells than the number of hook-shaped trap units to enable the cells to be trapped by the trap sites because of the relatively low possibility of capturing the cells. Therefore, it is difficult to pattern rare cells like circulating tumor cells (CTCs) and stem cells because of cell loss.

Some studies have designed trap structures along a main channel sequentially to capture single cells based on the principle of path of least flow resistance [27,28,29,30,31], and optimization of the geometric structures and flow field parameters was conducted to improve the single-cell capture efficiency. A recent study [32] proposed a single-cell trap structure which has an average single-cell capture efficiency of 90% on a large scale (100 × 100 trap sites). However, the cell density is relatively low, because there is a large inoperative space without trapped cells. In most designs, there is a compromise between trap site density and single-cell capture efficiency on a large scale.

To address the limitations mentioned above, a single-cell trap device was designed with high density, throughput, and efficiency; the device can be extended to a large-scale patterning structure. In this chip, numerous trap units were designed along the loop channel to trap the cells deterministically with high density and efficiency. Particularly, the U-shaped trap unit structure was separated into combinations of a groove and a slit; a theoretical model and a simulation model were established to investigate the optimized geometric parameters of the groove and slit. The density increased to 200 trap units in an area of 1 mm^2^, which is much better than that reported in some related studies [25,32]. Meanwhile, in this device, the cells can enter each trap unit in the order of the flow passage, which is also time sequential. The optimized device enables trapping of 500 cells within 1 min deterministically and sequentially, with up to 100% cell capture rate and 95% single-cell efficiency. For convenience of observation under a microscope, 10 × 10 trap sites were treated as a module that can be accommodated under a microscope field with a 20× objective lens. Through concurrent design, this device is capable of trapping 32,000 (320 modules) cells simultaneously within an area of 250 mm^2^.

## 2. Design and Theoretical Modeling

### 2.1. Structural Design

**Figure 1:**
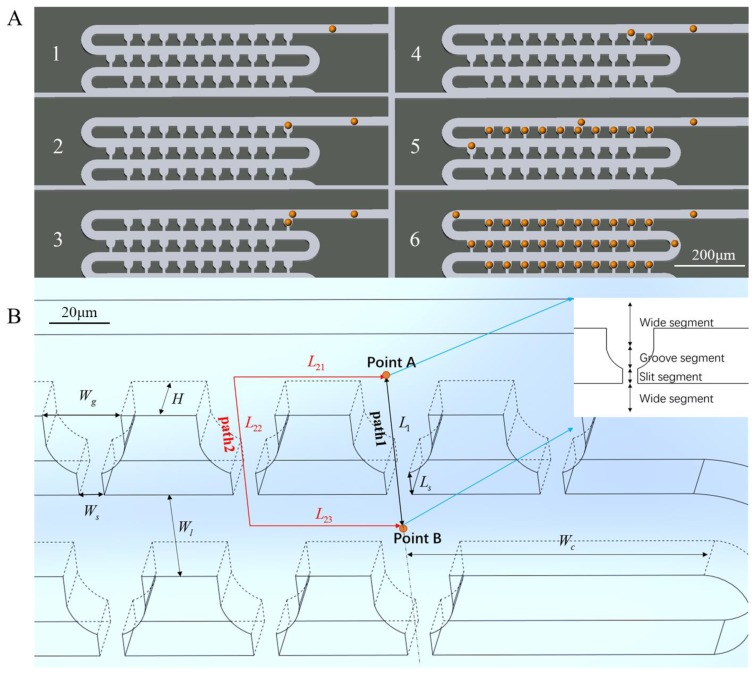
Microfluidic device for single-cell capture. (**A**) Schematic view of loop channel and trap units for hydrodynamic single-cell capture sequentially; (**B**) 3D view of trap units in the first row for theoretical and numerical analysis; the main geometric parameters are labeled. Each trap unit can be divided into four segments along the medial axis, and most of the pressure drop occurs along the narrow segment.

Figure 1A shows the schematic view of the single-cell trapping device designed in this study. The device is composed of a loop channel and thousands of aligned trap units, and each trap unit has a groove and a slit. The loop channel is used for the cell medium, while the grooves and slits form traps for the cells. This network structure enables each row of the device to be treated equally and independently, as it has row periodicity. Therefore, any random row can be chosen for the optimization of the geometrical parameters of the microfluidic channels to achieve cell trapping. A row with 10 trap units of a 10 × 10 array was selected for theoretical studies and simulations. For example, in two adjacent trap units in the first row in Figure 1B, there are two paths from point A to point B. Path 1 is a shortcut for the fluid through the groove and slit, while path 2 is longer than path 1.

Considering the path of least flow resistance in the channel, the flow resistance of the present trap unit should be less than that of the next one to achieve the sequential capture. Thus, the device was designed to ensure the trapping condition that the volume flow rate *Q*_1_ in path 1 is more than the flow rate *Q*_2_ in path 2; i.e., *RQ* (=*Q*_1_/*Q*_2_) > 1. In this condition, the cells in the flow will be carried into every trap unit sequentially from the first one to the last. Once the trap unit ahead is filled, its flow resistance will increase immediately. Therefore, the upcoming cells will be carried into the next trap unit. Once all the trap units are occupied, the cells in the flow will be redirected to the loop channels flowing into the next row. The above process is repeated, as shown in Figure 1A.

The device proposed here has three main advantages compared to the previously reported devices. First, this device has a very high density for single-cell patterning. The U-shaped trap units are designed so that they are distributed closely along the S-shaped loop channel, with a density of 200 trap units within an area of 1 mm^2^. The device can also be easily extended to achieve large-scale cell patterning, and a single-cell chip with 24,000 trap units was accomplished.

The second advantage is its high throughput and efficiency. It can trap cells fast, and the entire process of patterning of 1000 cells can be completed in 2 min, with up to 100% cell capture rate and 95% single-cell efficiency. Combined with the first advantage, this device can achieve single-cell patterning with a very high throughput.

Finally, the cells in the medium are trapped one at a time, which implies that the cells tend to be trapped until all the trap units are filled before they flow out of the device. Therefore, the combination of the S-shaped loop channels and U-shaped trap units can prevent the loss of several cells; this is important when manipulating some rare cells such as circulating tumor cells, circulating endothelial cells, minimal residual disease cells, and stem cells.

### 2.2. Theoretical Modeling

In this device, the maximum Reynolds number is sufficiently low for the flow to be considered as laminar. The Darcy-Weisbach equation and the solution to the Hagen-Poiseuille flow problem are applied to calculate the pressure drop in the channels [27,33]. The pressure difference in the channels can be expressed by the following equation [33].

(1) $$\Delta p=\frac{\rho V^{2}}{2}(f\frac{L}{D}+\sum K_{L})$$

where *f* is the friction factor, *ρ* is the fluid density, *V* is the average velocity, *L* is the channel length, *D* is the hydraulic diameter, and Σ*K_L_* represents the sum of minor losses due to the inlet, exit, and hydrodynamic development length.

For a rectangular channel, *D* can be expressed as 4*A*/*P*, and *V* can be expressed as *Q*/*A*, where *A* and *P* are the cross-sectional area and perimeter, respectively, of the channel; *Q* is the volumetric flow rate. The Darcy friction factor *f* is related to aspect ratio *α*, and the Reynolds number *Re* = *ρVD*/*μ*, where *μ* is the fluid viscosity. The aspect ratio is defined as either height/width or width/height, such that 0 ≤ *α* ≤ 1. The product of the Darcy friction factor and Reynolds number is a constant that depends on the aspect ratio, i.e., *f* × *Re* = *C*(*α*), and the friction constant *C*(*α*) [27,34] is dependent only on *α* for a fully developed laminar flow in rectangular channels.

Ignoring minor losses due to the inlet, exit, and hydrodynamic development length, etc., the expression for pressure difference can be obtained, after simplification, as follows.

(2) $$\Delta p=\frac{C(\alpha )}{32}\frac{\mu LQP^{2}}{A^{3}}$$

Referring to the simplified circuit diagram of the trap, the fluid can flow from junction A to junction B via path 1 or path 2. Equation (1) can be applied separately for path 1 and path 2. As the pressure drop is the same for both paths, the two expressions can be equated to yield the following result.

(3) $$\frac{Q_{1}}{Q_{2}}=\frac{\frac{C_{21}(\alpha_{21})L_{21}P_{21}^{2}}{A_{21}^{3}}+\frac{C_{22}(\alpha_{22})L_{22}^{\prime }P_{22}^{2}}{A_{22}^{3}}+\frac{C_{23}(\alpha_{23})L_{23}P_{23}^{2}}{A_{23}^{3}}}{\frac{C_{1}(\alpha_{1})L_{1}^{\prime }P_{1}^{2}}{A_{1}^{3}}}$$

where subscripts 1 and 2 represent paths 1 and 2, respectively. *L’*_1_ and *L’*_22_ are different from the lengths *L*_1_ and *L*_22_, because there are non-rectangular structures that include the groove and slit segments. For simplification, the pressure drop along the slit segment (*L_s_*) is used to denote the pressure drop along *L*_1_ and *L*_22_, because most of the pressure drop occurs along the narrow segment, which has the maximum constriction. From the relationships *A* = *W* × *H* and *P* = 2(*W* + *H*), where *H* is the height of the channels and *W* is the width of the corresponding cross-sectional area, the ratio of volume flow rates can be obtained.

(4) $$RQ=\frac{Q_{1}}{Q_{2}}=1+\frac{2C_{21}(\alpha_{21})L_{21}{\left(W_{l}+H\right)}^{2}W_{s}^{3}}{C_{s}(\alpha_{s})L_{s}{\left(W_{s}+H\right)}^{2}W_{l}^{3}}$$

where subscripts s and l represent the slit segment and loop channel, respectively. The second term is always positive, and hence *RQ* is greater than 1 for two adjacent trap units, which is consistent with the trap condition. It may be noted that the final expression for the flow rate ratio contains only geometric parameters. Therefore, this can be a simple and powerful tool to design and optimize the structure of the device, which can perform well at all velocities in the laminar flow regime.

### 2.3. Simulation Analysis

A 3D model, as shown in Figure 1B, was built using COMSOL Multiphysics 5.3a for laminar flow simulation to calculate *RQ*, so that the geometric parameters can be optimized to achieve a high trapping efficiency. In this simulation, the Jurkat cells (19.6 ± 4.0 μm) and MCF-7 cells (17.0 ± 5.0 μm) are treated as solid spheres. Therefore, both width *W*_l_ and height *H* of the loop channel are set to a constant value of 25 μm, which is a little larger than the biggest cells to avoid the device getting clogged. The width *W*_g_ and slit *W*_s_ of groove are the two variables affecting the volume flow rate ratio *RQ*. Considering the diameters of different cells, the default value of *W*_g_ and *W*_s_ can be specified as 25 μm and 4 μm, while Ws should be less than 10 μm; otherwise, the cells will squeeze through the trap unit because of their deformability.

**Figure 2:**
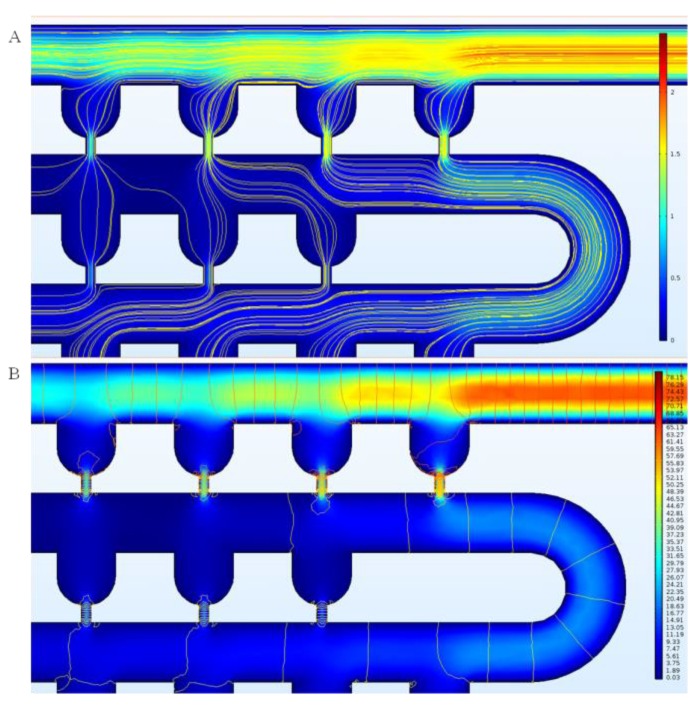
Simulation results of flow velocity field and pressure distribution in the device using the default geometric parameters, with an inlet flow rate of 3.75 × 10^−2^ μL/min. (**A**) Flow velocity field (mm/s), which is in the range of 0–2.5 mm/s; (**B**) Pressure distribution contour (Pa), which is in the range of 0–80 Pa.

With the default values of the parameters, the flow velocity field and pressure distribution in the device without any cell trapped can be obtained through simulation. The simulation results are shown in Figure 2. The rainbow colors represent the distribution of the flow velocity magnitude, and the streamlines indicate the flow direction. It can be seen that the flow moves faster through the slits, and that most of the pressure drop in the trap units occurs along the slit. The inlet flow rate of 1 mm/s (3.75 × 10^−2^ μL/min) in this case is relatively low. When the flow rate is increased to 100 mm/s (3.75 μL/min), no significant changes in the flow velocity field can be observed.

**Figure 3:**
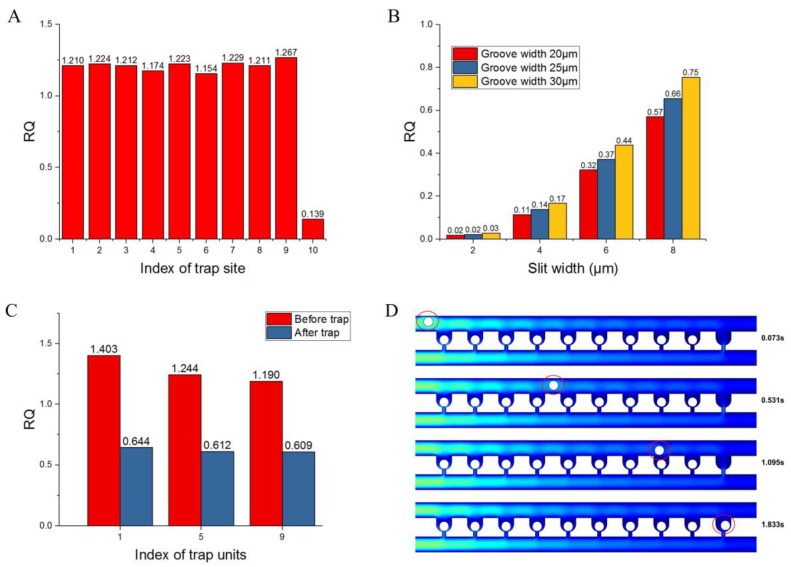
Simulation results of *RQ* values and single-cell dynamic trapping. (**A**) *RQ* values of trap units in the first row based on the default geometric parameters: *W*_l_ = 25 μm, *H* = 25 μm, *W*_s_ = 4 μm, and *W*_g_ = 25 μm; (**B**) *RQ* values of the last trap unit with different slit and groove widths; (**C**) *RQ* values of the first, fifth, and ninth trap units before and after trapping single cells; (**D**) Dynamic simulation to confirm the trapping result of the last trap unit when the previous nine trap units are occupied with cells.

The values of RQ for the 10 trap units in the first row were calculated for the condition when no cells are trapped; the results are shown in Figure 3A. It can be seen that there are some distinct characteristics of RQ. First, the last trap unit has the lowest value of RQ (<1). Second, all the RQ values for the first nine trap units exceed 1, which is consistent with the trapping condition. The RQ values of the previous 9 traps are compared with the next adjacent trap, while the 10th one is compared with the dc channel behind, so the last RQ value is lower the others. Because the cells are all close to the side of the groove when coming to the last capture unit, the RQ value does not need to be higher than 1 as in the previous ones. This implies that the cells will be carried into every trap unit one at a time from the first one to the last; this also indicates that the loop channel can be extended with more trap units between the first and the last one, and that their RQ values should exceed 1.

**Figure 4:**
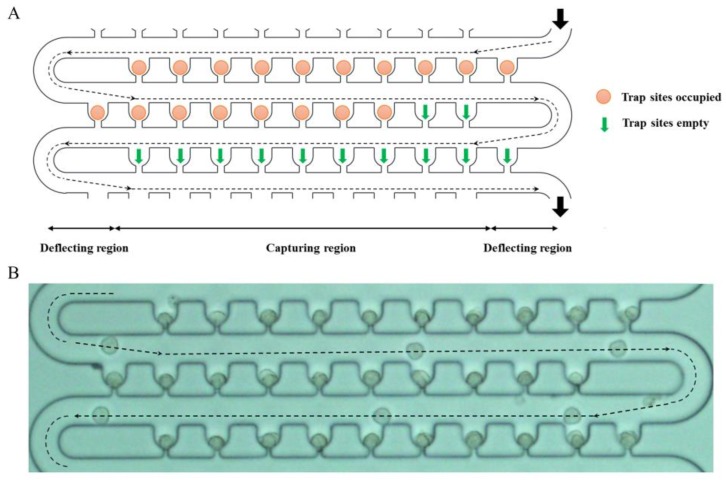
Cell trajectory pattern in the loop channel. (**A**) Schematic view of three rows of trap units showing the trajectory of the cells. The dotted lines and arrows represent the trajectory of the cells in the deflecting region and capturing region. (**B**) Merged image of a series of frames from a video showing the positions of a cell at different points of time during the experiment.

Based on the default values of the geometric parameters, when no cells are trapped, the influence of the variables *W*_g_ and *W*_s_ on *RQ* for the last trap unit was investigated, and the results are shown in Figure 3B. This result clearly shows that *RQ* has positive correlation with slit and groove width. When the slit width is 2 μm, the *RQ* values are almost 0, which shows that it is difficult to capture any cell. Even when the slit and groove widths are 8 μm and 30 μm, respectively, *RQ* is 0.75, which is still less than 1. The length *W*_c_ can be increased to improve the *RQ* value for the last trap unit according to the theoretical analysis given above, but it will cause a large decrease in the density of the trap units; this is a major advantage of this device. The cell trajectory in the loop channel has a distinct pattern, as shown in Figure 4A. Each periodic row contains a deflecting region and a capturing region. When the injected cells pass by a trap site, they will deflect to the side wall of the trap units in the deflecting region. If the trap unit is empty, a cell will be trapped. If the trap unit is occupied, the cells will move forward along the side wall of the trap units until all the empty trap units are filled. Figure 4B shows the positions of a cell at different points of time in the experiment, indicating the cell trajectory in the loop channel. Therefore, if the volume flow rate of the last trap unit is sufficiently large, the cell can still be trapped by the next trap site. In practice, the cells are distributed randomly when injected into the device from the inlet, which is favorable for their capture in the trap units. A relatively better value of *RQ*, i.e., greater than 0.24 (the ratio between the minimum MCF-7 cell radius of 6 μm and the width of 25 μm for the loop channel) can be chosen for the last trap unit; this implies that the slit width should be 6 μm or 8 μm. Furthermore, dynamic simulation is conducted to validate the results of trapping in the last trap unit when *RQ* = 0.37. The positions of a microsphere at different points in time are shown in Figure 3D. It can be inferred from the figure that a cell can still be trapped by the last trap unit when the trap units ahead are filled with cells.

Considering the cell diameter and actual experimental conditions, a slit width of 8 μm may be so large that some cells with relatively smaller diameters can squeeze through the trap unit because of their deformability. When the groove width is 30 μm, there is a strong possibility of each trap unit being occupied by two cells, which will decrease the single-cell trapping rate of this device. Thus, the final groove and slit dimensions after optimization were obtained as 25 μm and 6 μm, respectively. With the optimized geometric parameters, the RQ values of the first, fifth, and ninth trap units before trapping of cells were compared with the values after these trap sites were occupied with cells. Figure 3C clearly shows that the RQ values of all the three trap units are greater than 1 before they are filled with cells. After one trap unit trapped one cell, the RQ values of all these three trap units are below 1; this implies that there is insufficient fluidic flow to capture any cell, enabling single-cell capture with high efficiency.

## 3. Experiment and Analysis

### 3.1. Fabrication of the Device

The devices were fabricated using soft lithography. For SU-8 2075 mold fabrication, silicon wafers were rinsed in acetone, ethanol, and deionized water, and sonicated sequentially for 3 min in each liquid; the wafers were then baked on a hotplate at 100 °C for 5 min, followed by cooling down to room temperature. An SU-8 2075 negative photoresist (MicroChem Corp., Westborough, MA, USA) was spin-coated on the top of the clean silicon wafers to obtain a layer with a thickness of 25 μm, followed by prebaking (65 °C for 1 min and 95 °C for 5 min), UV exposure under a mask, postbaking (65 °C for 1 min and 95 °C for 5 min), development with SU-8 2075 developer, and hard baking (120 °C for 30 min). Negative molds of the microfluidic device were obtained and then treated with tridecafluoro-1,1,2,2-tetrahydrooctyl-1-trichlorosilane vapor (United Chemical Technologies, Bristol, PA, USA). For PDMS (Sylgard 184, Dow Corning Corporation, Midland, MI, USA) molding, the PDMS prepolymer and curing agent were uniformly mixed with a ratio of 10:1, and then poured onto the mold obtained above, followed by removal of air bubbles in the PDMS polymer with a vacuum pump and baking at 65 °C for 3 h. After the curing process, the PDMS polymer was peeled from the mold, and holes were punched at the entrance and exit of the channels. Finally, the PDMS polymer was bonded onto a glass slide using oxygen plasma for conducting experiments.

### 3.2. Cell Culture and Staining

In this work, Jurkat cell line (human peripheral blood leukocyte T cells) and MCF-7 cell line (human cervical carcinoma) were used. The Jurkat cells and MCF-7 cells were cultured with RPMI1640 (Thermo Fisher Scientific, Waltham, MA, USA) and Dulbecco’s modified Eagle’s medium (DMEM, Life Technologies, Carlsbad, CA, USA), respectively. Both cells were supplemented with 10% fetal bovine serum (FBS, Life Technologies, Carlsbad, CA, USA) and 1% Penicillin-Streptomycin (Sigma-Aldrich, St. Louis, MO, USA) under an environment of 37 °C and 5% CO_2_. Both cell line passages were conducted about every two days.

Before the experiment, the MCF-7 cells, as adherent cells, were detached from the culture dishes using 1 mL trypsin solution (Gibco, Gaithersburg, MD, USA), and incubated for 5 min at 37 °C. Once the cells were detached, DMEM was added to deactivate the trypsin in the solution, followed by centrifugation for 5 min at 1500 rpm. The top phase was removed, and the cells were resuspended in 1 × PBS solution with 2% Bovine serum albumin (BSA, Sigma-Aldrich, St. Louis, MO, USA) and 15% Optiprep (Sigma-Aldrich, St. Louis, MO, USA) to get the desired cell concentration of approximately 100,000 cells/mL. For the Jurkat cells, the process was the same, except that there was no detachment process. For fluorescent microscope observation, Hoechst 33,342 (Thermo Fisher Scientific, Waltham, MA, USA) was added into the cell suspension for DNA staining before loading the cells into the microfluidic devices.

### 3.3. Microfluidic System Operation

Before loading the cells, the microfluidic chips were primed with 1 × PBS solution with 2% BSA to remove the air in the channels. The cells in the suspension were injected into the device on a microscope scale with a syringe pump (70-3007, Harvard Apparatus, Holliston, MA, USA) at a speed of 0.5 μL/min. The single-cell trapping process and patterning results were observed with an inverted microscope (IX71, OLYMPUS, Tokyo, Japan), and fluorescent images were obtained via a blue fluorescent channel filter. To evaluate cell viability after the cells are trapped, a cell culture solution containing 0.4% trypan blue (Thermo Fisher Scientific, Waltham, MA, USA) was injected into the device to stain the cells directly.

### 3.4. Single-Cell Trapping

**Figure 5:**
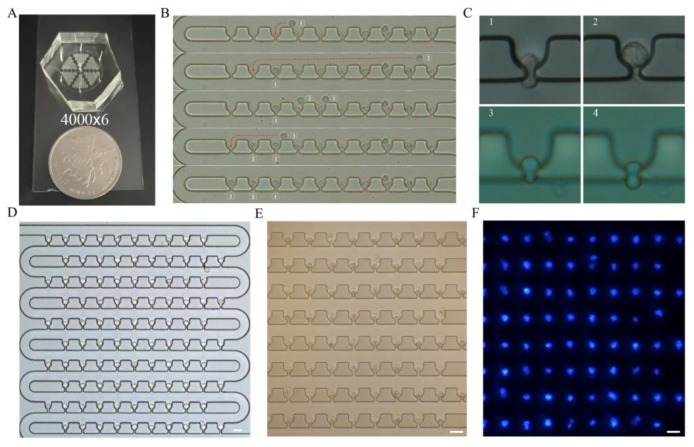
Single-cell trapping results (scale bar: 30 μm). (**A**) Image of soft lithography-fabricated microfluidic device with 100 × 10 trap units; (**B**) Five time-lapsed images showing the process of three MCF-7 cells trapped sequentially; (**C**) 1. Cell deformations at flow rates of 1 μL/min and 0.5 μL/min in 6 μm wide slit and 25 μm wide groove; 2. The process of a cell squeezing through an 8 μm wide slit at a flow rate of 0.5 μL/min; 3 and 4. The process that the cells push through the slit. (**D**,**E**) Bright-field microscopic images of trapped MCF-7 and Jurkat cells, respectively. (**F**) Fluorescent microscopic image of the same single-cell pattern of Jurkat cells shown in (**E**). Each blue-colored point represents a single cell.

The effectiveness of this microfluidic device was validated through experiments using Jurkat (19.6 ± 4.0 μm) and MCF-7 (17.0 ± 5.0 μm) cells. To achieve the highest single-cell pattern efficiency, microfluidic chips were fabricated through soft lithography, as shown in Figure 5A. The cells were injected using a microfluidic injection pump, and the inlet flow velocity was adjusted as required. The flow path of a cell to be trapped (Figure 4B) and the sequential single-cell trapping process for the upcoming cells were compared with the results predicted by the simulation (Figure 5B). The bright field images in Figure 5D,E show that the MCF-7 and Jurkat cells were captured well by the device with the 100 × 10 trap units. The fluorescent image of the Jurkat cells in Figure 5F further confirms the feasibility and effect of single-cell patterning in the device.

**Figure 6:**
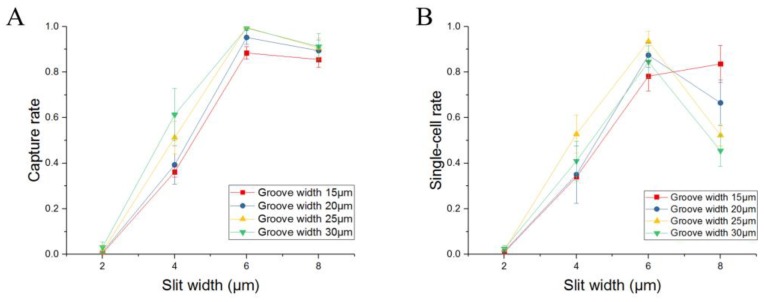
Experimental results of capture efficiency for various sizes of groove (15–30 μm) and slit (2–8 μm). (**A**) Capture efficiency; (**B**) Single-cell capture efficiency. The optimized sizes of the groove and slit are 25 μm and 6 μm, respectively, which enables up to 100% capture efficiency and 95% single-cell capture efficiency.

Devices with different geometric parameters had different values of trapping efficiency, as shown in Figure 6. For a constant groove width, when the slit width was increased from 2 μm to 6 μm, the capture rate increased, which is consistent with the simulation results. When the slit width was 8 μm, the capture rate decreased slightly, as cells with smaller sizes can squeeze through the slit because of their deformability under high flow rates, as shown in Figure 5C (3). The groove width had a significant effect on single-cell trapping rate when the width was 30 μm, because it had a higher possibility of trapping more than one cell. Therefore, the optimal sizes of the groove and slit should be chosen as 25 μm and 6 μm, respectively. In summary, the device with optimized geometric parameters had almost 100% cell capture rate and more than 90% single-cell capture rate for Jurkat and MCF-7 cells patterning. Furthermore, the experiments indicated the robustness of the device for capturing different cells with sizes varying from 12 μm to 23.6 μm. Furthermore, the channel will be blocked when the cell concentration is too high. Therefore, we usually set it between 10^4^/mL and 10^5^/mL in the experiments.

Flow rate has an influence on trap efficiency. When the flow rate is approximately 0.01 μL/min, it takes more time to inject the cells into the device, and the cells tend to settle in the inlet reservoir, resulting in cell loss. At a flow rate of 0.1 μL/min, there will be some reduction in the time to inject the cells, and the whole process of capturing 1000 cells in the trap units could be accomplished in 2 min using the optimized device; this is faster than the results obtained with other devices working on a similar principle [25,35] (200 trap sites in 30 min and 400 trap sites in 10 min). In this device, single cells can be trapped well without significant deformation because of low shear stress, as shown in Figure 5C (2); this causes less damage to cell viability. The viability of the cells was tested after they had been trapped for approximately 30 min with a cell culture solution containing 0.4% trypan blue injected into the device to stain the cells directly; the viability obtained was 98%. For flow rates above 1 μL/min, and even up to 10 μL/min, the high shear stress at the slit segment can sometimes squeeze the MCF-7 cells through the trap unit with significant deformation, as shown in Figure 5C (1). The high shear stress at the slit segment will cause severe mechanical damage to the trapped cells and decrease cell viability significantly. Therefore, a flow rate of 0.1–0.5 μL/min is recommended for low cell loss and relatively high single-cell capture efficiency.

The device aims to isolate single cells quickly and accurately, which are not needed to stay for a long time, reducing the effect of the porosity of the PDMS. Based on this chip, single-cell analysis experiments could be conducted. Also, they can be transferred to the pore plate for further study, such as the study of heterogeneities based on drug stimuli. This chip is reusable, in theory. After capturing the cells, cells could be drained out by injecting the fluid into the chip using a microfluidic injection pump. After cleaning, it can be used again for the next experiment. However, the process of the chip fabrication is relatively simple, mainly including PDMS molding using Si wafer after photoetching. Therefore, in practice, we do not reuse the chip.

## 4. Conclusions

This paper presents a microfluidic device for single-cell capture with enhanced loading efficiency, high capture speed, and high density of patterning. A theoretical model was established and dynamic simulation was conducted to optimize the structure and capture efficiency. The optimized device can trap 1000 cells deterministically in 2 min, with up to 100% capture efficiency and 95% single-cell capture efficiency in a 5 mm^2^ area. We randomly selected sixteen views to count the traps captured cells and single cells under the microscope, each with an array of about 10 × 10, and then averaged them to get the capture rate and the single-cell rate. This device has higher throughput, efficiency, and density compared with other microfluidic trapping devices. We compared the methods mentioned in other references in terms of throughput, capture rate, and single-cell rate, among which our device had the highest cell capture rate and the highest efficiency. There was one method for single-cell capture whose capture rate was higher than that of our device, whereas its cell damage rate was high, and its speed was very slow [29]. As the cells are trapped sequentially, cell loss will be reduced, which is crucial for manipulating some rare cells. Using this device, the response of 100 cells can be observed simultaneously in time-lapse experiments under a microscope field with a 20× objective lens; this enables the investigation of single-cell heterogeneity by exposing the cell to various chemical stimuli. The cells can also be recovered by reversely infusing solutions for further cellular assay. With excellent features, this device has great potential in important studies on cell heterogeneity, drug toxicity, and manipulation of rare cells such as circulating tumor cells and stem cells.
